# Retraction: Yu et al. Low Iron Diet Increases Susceptibility to Noise-Induced Hearing Loss in Young Rats. *Nutrients* 2016, *8*, 456

**DOI:** 10.3390/nu9040422

**Published:** 2017-04-25

**Authors:** Nutrients Editorial Office

**Affiliations:** MDPI AG, St. Alban-Anlage 66, 4052 Basel, Switzerland; nutrients@mdpi.com

The *Nutrients* Editorial Office has recently been made aware that the figures in the title paper [1] are taken from the same micrographs as those of other papers by the same authors, but purporting to show different species. In particular, Figure 2D of [1] (rat) is identical to Figure 2D of [2] (mouse), Figure 4B of [1] (rat) is identical to Figure 4B of [3], and Figure 5 of [1] is identical to Figure 3B of [2]. Given this, we do not have confidence in the figures and thus the conclusions of the paper. In order to correct the publication record, the paper [1] will be marked as retracted. 

*Nutrients* is a member of the Committee on Publication Ethics (COPE) and strives to uphold the highest ethical standards; misuse of images is not acceptable and we are committed to taking appropriate action when such cases are reported. We apologize to readers of *Nutrients* and wish to thank the reader who first reported this case.
